# SA-MTP: a structure-aware framework for multifunctional therapeutic peptide annotation

**DOI:** 10.1093/bib/bbag361

**Published:** 2026-07-03

**Authors:** Wenping Yu, Zhewen Li, Wei Xu, Yu Zhao, Nan Sun

**Affiliations:** College of Artificial Intelligence, Tianjin University of Science and Technology, No. 9, 13th Avenue, Binhai New Area, Tianjin 300457, China; College of Artificial Intelligence, Tianjin University of Science and Technology, No. 9, 13th Avenue, Binhai New Area, Tianjin 300457, China; College of Artificial Intelligence, Tianjin University of Science and Technology, No. 9, 13th Avenue, Binhai New Area, Tianjin 300457, China; College of Artificial Intelligence, Tianjin University of Science and Technology, No. 9, 13th Avenue, Binhai New Area, Tianjin 300457, China; Beijing Institute of Mathematical Sciences and Applications (BIMSA), No. 544, Hefangkou Village, Huaibei Town, Huairou District, Beijing 101408, China; Yau Mathematical Sciences Center, Tsinghua University, No. 30 Shuangqing Road, Haidian District, Beijing 100084, China

**Keywords:** therapeutic peptides, multi-label prediction, structure-aware learning, protein language models, graph attention network

## Abstract

Therapeutic peptides show many biological activities and are now widely viewed as promising candidates for new drug development. Accurate functional annotation of therapeutic peptides is still difficult. This difficulty comes from their short sequence length, strong structural flexibility, and the presence of multiple biological functions within a single peptide.Here, we introduce Structure-Aware Multi-Label Therapeutic Peptide Predictor (SA-MTP), a structure-aware framework designed for multifunctional annotation of therapeutic peptides. SA-MTP combines pretrained protein language models with a graph attention network to capture sequence semantics and probabilistic structural features. Input-dependent structure-aware graphs are constructed to describe conformational variation, which is especially common in short peptides. Benchmarking experiments across 15 therapeutic function categories were conducted using datasets. The results show that SA-MTP achieves better performance than existing methods across several evaluation metrics, including accuracy, F1-score, and Matthews correlation coefficient.

## Introduction

Therapeutic peptides (TPs) are bioactive molecules made of short amino acid chains. They combine advantages of small-molecule drugs and protein-based therapeutics, including high specificity, low immunogenicity, good biodegradability, and simple synthesis [1, 2]. Because of these properties, TPs have been widely studied for antimicrobial, anticancer, anti-inflammatory, antiviral, antioxidant, and immunomodulatory uses. Advances in peptide synthesis, screening, and modification have promoted peptide-based therapeutics as an important pharmaceutical form alongside small molecules and antibodies [3]. Accurate functional annotation and activity prediction of TPs has therefore become a key problem in bioinformatics and peptide-based drug discovery.

Experimental studies show that many TPs display more than one biological activity rather than a single function. These multifunctional therapeutic peptides (MTPs) may inhibit tumor cell growth while also showing antimicrobial or immunomodulatory activity [4]. Multifunctionality offers benefits in drug development, including reduced side effects compared with combination therapies [5] and multi-target regulation in complex diseases such as cancer, viral infections, and inflammatory disorders [6]. This property also creates difficulties for computational modeling. Many existing prediction methods fail to represent functional diversity and the complex relationships among multiple activities in short peptide sequences. The development of bioinformatics methods that identify and describe MTP functions [7] remains important for peptide screening, rational design, and understanding peptide function across biological pathways.

High-throughput peptide sequencing technologies have developed rapidly. Public databases such as APD3, dbAMP, and DRAMP 2.0 have been constructed. Large collections of peptide sequences and functional annotations have been accumulated. These resources support computational prediction of MTPs [8–10]. Many computational methods have been proposed based on these datasets. These methods include traditional machine learning models, deep learning approaches, and pretrained model based frameworks.

Early studies focused on single therapeutic activities. Prediction tasks were framed as binary classification problems. These methods relied on manually designed sequence features. These features include amino acid composition, dipeptide composition, pseudo amino acid composition, physicochemical properties, and position related features [11, 12]. Models such as AntiCP, iACP, and related variants showed stable performance in specific tasks such as anticancer peptide prediction. This effect was stronger when training data were limited [13–15]. More general frameworks such as PEPred-Suite and PPTPP extended this approach to multiple therapeutic categories. These frameworks combined different feature types and used ensemble learning [11, 12]. At the same time, early multi-label models such as MLAMP explicitly considered functional co-occurrence and allowed simultaneous prediction of multiple peptide activities [16]. Despite their reported performance, these approaches remain limited by their dependence on low-order statistical features and their insufficient ability to represent higher-order residue interactions and complex semantic relationships among functional labels.

Later progress in deep learning shifted the focus to end-to-end feature learning directly from raw peptide sequences. These approaches used convolutional networks, recurrent networks, or hybrid neural structures. Models such as DeepACP, ACP-MHCNN, and xDeep-AcPEP showed that deep architectures can automatically learn useful local and contextual patterns and often perform better than traditional machine learning methods in peptide function prediction [17–19]. In multi-label scenarios, models including MLBP and MultiPep further applied deep learning to predict several therapeutic functions at the same time. These studies demonstrated the value of shared representations and multitask learning when modeling complex functional combinations [20, 21]. More recent work has investigated graph attention mechanisms and multimodal feature fusion, indicating that graph-based representations can help capture structural and functional dependencies in MTP prediction [22, 23]. In many existing studies, structural information is still used in a static or auxiliary way. This design limits the ability of these models to represent the conformational variability that is common in short peptides.

Unlike globular proteins with relatively stable tertiary conformations, many TPs are short, flexible, and partially disordered, often exhibiting substantial conformational heterogeneity under physiological conditions. In such cases, a single predicted static structure may not fully capture the range of accessible conformational states associated with peptide activity. Probabilistic structural tendencies and residue-level contextual dependencies may therefore provide more robust and scalable representations for TP modeling. Motivated by this observation, recent studies have increasingly explored lightweight structure-aware strategies based on sequence-derived structural priors and protein language model (PLM) representations rather than relying exclusively on explicit 3D coordinates.

With the development of PLMs, newer MTP prediction frameworks increasingly rely on large-scale pretrained contextual embeddings. These embeddings are often combined with attention mechanisms and additional structural signals. Models such as LMPred and PeptideBERT demonstrated that PLM-derived representations substantially outperform handcrafted features across a range of peptide-related tasks [24, 25]. In TP prediction, TPpred-ATMV, TPpred-LE, and TPpred-SC progressively incorporated multi-view learning, label embeddings, and structural constraints to improve modeling of functional correlations and structure–function relationships [26–28]. Recent methods, including AMHF-TP and METFAN, further integrate PLM embeddings with attention-based fusion of physicochemical and external features, achieving state-of-the-art performance on multiple MTP benchmarks [6, 29]. Nevertheless, effectively modeling the interplay between sequence semantics, structural heterogeneity, and multi-label functional dependencies in short TPs remains an open challenge.

Despite substantial advances in feature representation learning and predictive accuracy, existing computational methods for MTP prediction continue to face several fundamental challenges.

- **Insufficient utilization of structural information.** Although recent studies have begun to incorporate structural descriptors, most models rely on single, static representations or fixed-topology sequence graphs, thereby overlooking the probabilistic and dynamic nature of peptide conformations. Experimental and structural studies have demonstrated that many antimicrobial and TPs are intrinsically disordered in aqueous environments and adopt ordered conformations only upon interaction with membranes or molecular targets [30–32]. In particular, antimicrobial activity often depends on an ensemble of accessible conformational states rather than a single stable fold [30], and disorder-to-order transitions upon target binding are widely observed [33]. Reducing such conformational heterogeneity to static descriptors obscures local structural cues and weakens modeling of sequence–structure–function relationships.
- **Limited model interpretability.** Most existing approaches primarily rely on attention visualization or embedding similarity, while explicit integration of residue-level structural priors for functional interpretation remains relatively limited [34]. The lack of structure-aware interpretability restricts the utility of current models for mechanistic inference and rational peptide design [35].
- **Suboptimal generalization performance.** Many models are prone to overfitting when confronted with structurally complex peptides, conformational uncertainty, or long-tailed functional label distributions, resulting in unstable performance across functional categories and limited transferability to unseen sequences [36, 37].

To address these limitations, we propose Structure-Aware Multi-Label Therapeutic Peptide Predictor (SA-MTP), a structure-aware framework for multi-label TP prediction. SA-MTP integrates pretrained PLMs, input-dependent graph construction, and label-aware classification within a unified sequence–structure–function framework. The model combines probabilistic secondary-structure distributions predicted by PSIPRED with PLM-derived residue–residue contact priors to construct uncertainty-aware structural graphs that capture conformational tendencies and residue-level structural dependencies without relying on explicit experimentally resolved 3D structures.

## Materials and methods

### Dataset and preprocessing

We adopt the MTP benchmark dataset released by TPpred-LE and strictly follow its preprocessing protocol to ensure fair and reproducible comparison with existing methods [27]. Sequence redundancy is removed using CD-HIT with a 90% identity threshold, after which the dataset is divided into training, validation, and independent test sets using an 8:1:1 split. The distributions of the 15 TP categories across the three subsets are summarized in Supplementary Methods E1. To evaluate generalization under stricter homology constraints, an additional low-homology benchmark was constructed using CD-HIT with a 40% sequence identity threshold. Details of the low-homology evaluation protocol are provided in Supplementary Methods E2.

TPs are usually short and highly flexible, often showing strong conformational uncertainty in solution. To capture this characteristic, probabilistic secondary-structure features in SS2 format are generated using PSIPRED [38]. For each residue, PSIPRED produces a probability distribution over three structural states—$\alpha $-helix (H), $\beta $-strand (E), and coil (C)—providing an uncertainty-aware structural representation that reflects the ensemble nature of peptide conformations and supports structure-aware modeling.

For sequence representation, residue level embeddings are obtained using the pretrained PLM ESM-2 with 650M parameters [39]. To reduce computational cost and improve model stability, the embeddings are projected and normalized during preprocessing. Additional details on SS2 feature generation and embedding preprocessing are given in Supplementary Methods S1.

### Overview of SA-MTP model

**Fig. 1.**
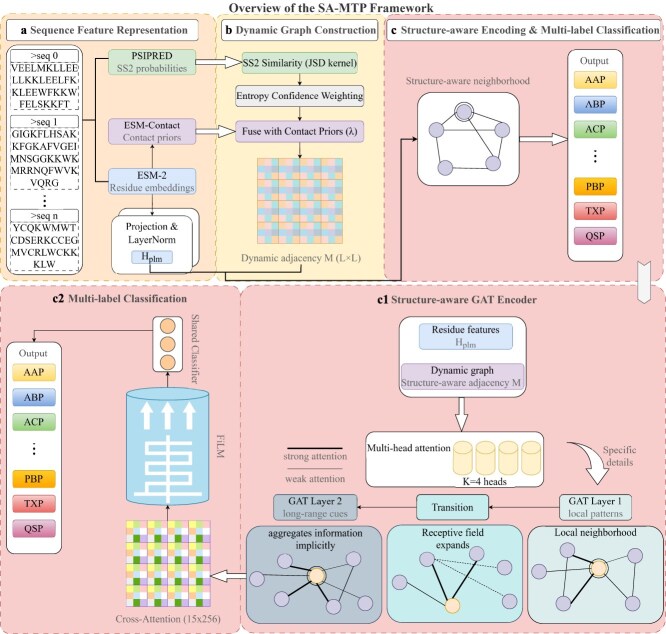
Overview of the SA-MTP framework.

The proposed SA-MTP combines sequence meaning and flexible structure signals to support comprehensive functional profiling of TPs. The framework comprises four key components: a sequence representation module, an input-dependent dynamic graph construction module, a structure-aware graph attention encoder, and a label embedding–driven classification module. An overview of the complete workflow is illustrated in Fig. 1.

Given an input peptide sequence, residue-level semantic representations are first extracted using the pretrained PLM ESM-2, followed by linear projection and normalization to obtain compact sequence embeddings. These embeddings are then jointly modeled with probabilistic structural information through the core structure-aware graph attention encoder, enabling dynamic integration of sequence and structure at the residue level. Finally, a Feature-wise Linear Modulation (FiLM)-based classification head performs label-specific feature modulation to account for functional heterogeneity across therapeutic categories and outputs probabilistic predictions for 15 therapeutic functions. An adaptive thresholding strategy is subsequently applied to generate the final multi-label outputs.

#### Sequence feature representation module

In SA-MTP, sequence-level semantic representations are extracted using the pretrained PLM ESM-2, which encodes rich contextual dependencies and implicitly captures evolutionary and structural signals at the residue level [39, 40]. For a peptide sequence of length $L$, ESM-2 generates a sequence of residue embeddings that serve as informative initial features for downstream modeling, particularly suited for short and functionally diverse TPs.

To match the input dimensionality of the structure-aware graph encoder and mitigate feature redundancy, the ESM-2 embeddings are projected into a compact latent space using a lightweight projection network incorporating normalization and dropout. The resulting residue-level representation matrix is subsequently used as the initial node feature input to the dynamic graph attention encoder, providing the sequence-semantic basis for joint sequence–structure modeling. Detailed network architectures and projection-layer specifications are provided in Supplementary Methods S2.

#### Dynamic graph structure modeling module

TPs generally exhibit highly flexible 3D conformations, with local structural states strongly influenced by sequence context, solvent conditions, and external perturbations, resulting in substantial conformational heterogeneity [41]. SA-MTP builds an input-dependent dynamic structure-aware graph that directly combines probabilistic secondary-structure signals with predicted residue–residue contact priors. The generated weighted adjacency matrix reflects local conformational similarity and long-range structural relations, acting as the structural basis for later graph attention encoding.

At the residue level, secondary-structure probability distributions are used to form a local structural similarity matrix. This matrix is adjusted using confidence weights to reflect prediction uncertainty in flexible or disordered regions. Global structural context is introduced by merging the confidence-weighted local similarity with residue–residue contact probabilities produced by ESM-2. The resulting dynamic adjacency matrix is defined as 


(1) \begin{align*}& M_{\mathrm{dyn}}=(1-\lambda)M_{\mathrm{ss}}+\lambda C,\end{align*}

where $M_{\mathrm{ss}}$ denotes the confidence-weighted secondary-structure similarity matrix, $C$ represents the predicted contact probability matrix, and $\lambda $ controls the balance between local conformational similarity and long-range contact priors. Unless otherwise specified, $\lambda = 0.25$, which consistently achieved strong overall performance in the hyperparameter sensitivity analysis. SA-MTP remained stable across a broad range of $\lambda $ values, indicating limited sensitivity to precise fusion parameter selection.

To reduce noise and computational cost, the dynamic graph is sparsified by retaining only the most informative neighbors for each residue based on $M_{\mathrm{dyn}}$: 


(2) \begin{align*} & N_{k}(i) = \mathrm{Top-}k_{j} \, M_{\mathrm{dyn}}[i,j], \end{align*}

(3) \begin{align*} & k=\min(10,L-1), \end{align*}

where $k = min(10,L-1)$, which consistently provided a favorable balance between graph connectivity and noise reduction in the hyperparameter sensitivity analysis. SA-MTP exhibited robust performance across different neighborhood sizes. Section Hyperparameter sensitivity analysis provides a detailed analysis of the hyperparameter sensitivity experiment and its observed effects. Graph symmetrization is subsequently applied to ensure reciprocal connectivity. During training, DropEdge regularization is introduced to improve robustness and generalization, whereas during inference the full adjacency matrix is used to fully exploit structural information. Detailed formulations of structural similarity computation, uncertainty modeling, graph sparsification, and regularization strategies are provided in Supplementary Methods S3.

#### Graph attention encoding module

After obtaining the residue-level sequence representations and the dynamic structural graph, SA-MTP feeds the projected feature matrix $H_{\mathrm{plm}} \in \mathbb{R}^{L \times 256}$ together with the dynamic weighted adjacency matrix $M \in \mathbb{R}^{L \times L}$ into a graph attention network (GAT) to achieve deep integration of sequence semantics and structure-dependent interactions. Each residue is treated as a node in the graph, while the dynamic adjacency matrix defines structure-aware neighborhood constraints.

The GAT uses learnable attention to flexibly collect information from nearby and distant residues. This process follows the dynamic structural graph [42, 43]. For a residue node $i$, normalized attention weights within its structure-aware neighborhood $N(i)$ are calculated as 


(4) \begin{align*} &\alpha_{ij}^{(l)} = \frac{\exp\left(e_{ij}^{(l)}\right)}{\sum\limits_{k \in \mathcal{N}(i)} \exp\left(e_{ik}^{(l)}\right)},\nonumber\\ &\quad\qquad j \in N(i),\end{align*}

where $e_{ij}^{(l)}$ denotes the unnormalized attention score associated with residue pair $(i,j)$ at layer $l$. The representation of node $i$ is then updated via weighted aggregation: 


(5) \begin{align*}& h_{i}^{(l+1)} = \sigma\left( \sum_{j \in \mathcal{N}(i)} \alpha_{ij}^{(l)} W^{(l)} h_{j}^{(l)} \right),\end{align*}

where $W^{(l)}$ is a learnable projection matrix and $\sigma (\cdot )$ represents the GELU activation function.

To capture multi-scale structural dependencies, SA-MTP employs a two-layer stack of multi-head GAT modules that progressively integrate local conformational patterns and long-range structural interactions. Residual connections and dropout are applied to stabilize training and improve generalization. After graph attention encoding, the model produces the final structure-aware residue representation $H_{\mathrm{gat}} \in \mathbb{R}^{L \times 256}$, which serves as input to the subsequent label-embedding and FiLM-based classification module. Detailed formulations of multi-head attention, residual connections, and regularization strategies are provided in Supplementary Methods S4.

#### Label embedding and classification module

To capture latent semantic dependencies among therapeutic functions and enhance discriminative capacity in the multi-label output space, SA-MTP augments the structure-aware sequence representation $H_{gat}$ with a label-aware classification architecture that integrates learnable label embeddings, sequence–label interaction, and FiLM.

Given $C=15$ therapeutic function labels, we define a learnable label embedding matrix: 


(6) \begin{align*}& E_{\mathrm{label}} = \begin{bmatrix} e_{1}^\top \\ e_{2}^\top \\ \vdots \\ e_{C}^\top \end{bmatrix} \in \mathbb{R}^{C \times 256},\end{align*}

where each row $e_{i}$ represents the semantic embedding of the $i$ therapeutic function. These embeddings enable explicit modeling of label semantics and facilitate label-specific interaction with the structure-aware residue representations.

To obtain label-conditioned peptide features, a cross-attention mechanism is employed in which each label embedding attends over the residue representations in $H_{\mathrm{gat}}$. For label $i$, a label related representation is computed as 


(7) \begin{align*}& z_{i} = \mathrm{Attn}(e_{i}, H_{\mathrm{gat}}).\end{align*}

$Attn(\cdot )$ is an attention-based aggregation step. It assigns weights to residues based on relevance to the functional label. This design allows each therapeutic function to focus on peptide regions with meaningful structural and semantic information.

To model functional differences in a direct way while keeping the classification head simple, SA-MTP uses FiLM to apply label-specific feature modulation. For each label $i$, a modulation vector $s_{i}\in (0,1)^{256}$ is produced and applied through channel-wise scaling: 


(8) \begin{align*}& z_{i} = (1 + \alpha s_{i}) \odot z_{i},\end{align*}

where $\alpha $ sets the strength of modulation and $\odot $ represents element-wise multiplication. This mechanism enables different therapeutic functions to emphasize distinct structural and semantic cues while sharing a common backbone representation, thereby improving parameter efficiency and generalization.

Finally, all modulated label-specific representations are passed through a shared linear classifier with sigmoid activation to produce the final multi-label prediction vector $y\in [0,1]^{C}$, where each element represents the predicted probability of the corresponding therapeutic function. Detailed formulations of the attention operation and FiLM parameterization are provided in Supplementary Methods S5.

#### Threshold optimization strategy

In multi-label prediction tasks, class distributions are often highly imbalanced, rendering a uniform decision threshold (e.g. 0.5) suboptimal. Such fixed thresholds tend to suppress recall for rare labels and consequently degrade balanced performance metrics such as the F1-score and Matthews correlation coefficient (MCC) [44].

To address this issue, SA-MTP adopts a class-specific, performance-driven adaptive thresholding strategy, in which an optimal decision threshold is independently determined for each therapeutic function label based on validation performance. Specifically, for each label, a set of candidate thresholds is evaluated on the validation set, and the threshold that maximizes a target metric (F1-score or MCC, depending on label characteristics and prevalence) is selected.

During inference, the optimized threshold is applied to the predicted probability of each label to obtain the final binary decision: 


(9) \begin{align*}& y_{t}^{(c)} = \mathrm{I}\left(y^{(c)} \geq t_{c}^{\ast}\right),\end{align*}

where $t_{c}^{\ast }$ denotes the optimized threshold for label $c$, $y_{t}^{(c)}$ is the predicted probability, and $I(\cdot )$ is the indicator function. A detailed analysis of the threshold selection process and its observed effects is presented in Section Threshold optimization.

This adaptive thresholding strategy reduces the influence of label imbalance, improves recall for rare therapeutic functions, and produces more balanced and well-calibrated predictions across functional categories.

### Experimental setup and evaluation metrics

#### Experimental setup

Model training used the AdamW optimizer with an initial learning rate of $1 \times 10^{-4}$ and a weight decay of $1 \times 10^{-5}$. The batch size was 32 and training ran for up to 100 epochs. GELU served as the activation function and a dropout rate of 0.1 was applied across the network. Binary cross-entropy loss with logits was used for multi-label optimization.

Model training used the AdamW optimizer with an initial learning rate of $1 \times 10^{-4}$ and a weight decay of $1 \times 10^{-5}$. The batch size was 32, and training was performed for up to 50 epochs. GELU was used as the activation function, and a dropout rate of 0.1 was applied throughout the network. Binary cross-entropy loss with logits was used for multi-label optimization.

A two-stage training strategy was adopted to improve optimization stability and support label-aware modulation. In the first stage, the sequence projection layers, graph construction modules, and GAT encoder were frozen, while only the FiLM parameters and the final classifier were updated. During the second stage, all modules were unfrozen to enable joint optimization of sequence, structure, and label representations.

All parameters were initialized using Xavier uniform initialization. Random seeds were fixed to ensure reproducibility. Early stopping was applied based on validation performance, and training was terminated after 15 epochs without improvement.

#### Evaluation metrics

To assess SA-MTP performance on the multi-label peptide function prediction task, both label-level and sample-level metrics were evaluated.

At the label level, AUC, MCC, and F1-score were reported for each therapeutic function. These metrics reflect discrimination capability, robustness to class imbalance, and precision–recall balance.

At the sample level, precision, recall, and sample-level F1-score were used to evaluate overall prediction quality for each peptide.

All metrics were computed on the independent test set. Five runs with different random splits were performed and mean results were reported.

## Results and discussion

### Performance comparison of SA-MTP with existing methods

We evaluated the effectiveness of the proposed structure-aware modeling strategy in SA-MTP using the benchmark dataset and evaluation protocol established by TPpred-LE. Both label-level and sample-level performance were examined. For label-level metrics, including AUC, MCC, and F1-score, SA-MTP was compared with the best reported results from representative state-of-the-art methods [11, 12, 26, 27]. For sample-level precision and recall evaluation, several publicly available baseline methods were reimplemented and evaluated under the same preprocessing and experimental settings to obtain comparable global performance estimates [21, 27, 45–49].

**Fig. 2.**
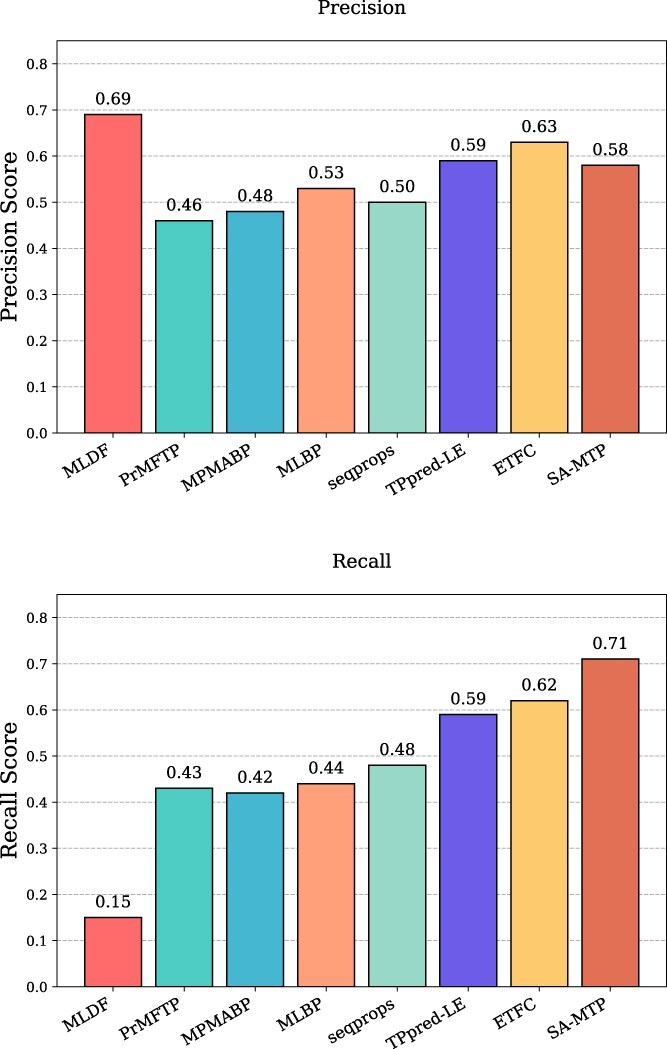
Overall performance comparison.

As summarized in Table 1 and Fig. 2, SA-MTP outperforms existing methods across all reported label-level metrics, including AUC, MCC, and F1-score. It also achieves higher overall precision and recall at the sample level. The improvement is more evident for functional classes with strong label imbalance or complex structural patterns. This result reflects the benefit of using structure-aware modeling for MTP prediction.

**Table 1:** Performance comparison of different methods across peptide functions

**Function**	**Method**	**AUC**	**MCC**	**F1**
AAP	PEPred-Suite^a^	0.577	0.02	0.03
	PPTPP^a^^,^^b^	0.604	0.037	0.033
	TPpred-ATMV^a^	0.583	0.009	0.027
	TPpred-LE	0.745	0.278	0.285
	SA-MTP	**0.807$\pm $0.026**	**0.351$\pm $0.092**	**0.340$\pm $0.093**
ABP	PEPred-Suite^a^	0.744	0.261	0.367
	PPTPP^a^^,^^b^	0.732	0.261	0.365
	TPpred-ATMV^a^	0.731	0.256	0.36
	TPpred-LE	0.834	0.337	0.426
	SA-MTP	**0.843$\pm $0.016**	**0.433$\pm $0.035**	**0.506$\pm $0.026**
AIP	PEPred-Suite^a^	0.363	−0.19	0.18
	PPTPP^a^^,^^b^	0.386	−0.06	0.168
	TPpred-ATMV^a^	0.369	−0.25	0.196
	TPpred-LE	0.895	0.527	0.594
	SA-MTP	**0.920$\pm $0.009**	**0.586$\pm $0.014**	**0.649$\pm $0.012**
AVP	PEPred-Suite^a^	0.382	−0.129	0.147
	PPTPP^a^^,^^b^	0.404	−0.11	0.169
	TPpred-ATMV^a^	0.394	−0.118	0.135
	TPpred-LE	0.835	0.457	0.529
	SA-MTP	**0.847$\pm $0.005**	**0.508$\pm $0.009**	**0.564$\pm $0.010**
CPP	PEPred-Suite^a^	0.813	0.152	0.142
	PPTPP^a^^,^^b^	0.814	0.14	0.139
	TPpred-ATMV^a^	0.815	0.152	0.139
	TPpred-LE	0.899	0.477	0.502
	SA-MTP	**0.910$\pm $0.013**	**0.526$\pm $0.043**	**0.546$\pm $0.043**
PBP	PEPred-Suite^a^	0.907	0.153	0.069
	PPTPP^a^^,^^b^	0.829	0.119	0.07
	TPpred-ATMV^a^	0.836	0.153	0.086
	TPpred-LE	0.934	0.443	0.430
	SA-MTP	**0.964$\pm $0.01**	**0.517$\pm $0.134**	**0.492$\pm $0.119**
QSP	PEPred-Suite^a^	0.835	0.113	0.043
	PPTPP^a^^,^^b^	0.815	0.08	0.033
	TPpred-ATMV^a^	0.772	0.054	0.027
	TPpred-LE	0.879	0.420	0.391
	SA-MTP	**0.955$\pm $0.033**	**0.467$\pm $0.170**	**0.448$\pm $0.166**

^a^The results are obtained by running their standalone programs.

^b^PPTPP contains three variant approaches, including PPTPP-cls, PPTPP-prb, and PPTPP-fus, among which only the best results for each metric are reported. Adapted from Table 2 of Lv H, Yan K, and Liu B. TPpred-LE: therapeutic peptide function prediction based on label embedding. BMC Biology 21, 238 (2023), licensed under CC BY 4.0; modified by adding SA-MTP results [27].

Further analysis of precision and recall shows clear differences between methods. MLDF reaches the highest precision with a value of 0.69. Recall remains very low at 0.15. This pattern reflects a conservative prediction style. High confidence positives are favored. Many relevant functions are missed. SA-MTP shows a more balanced pattern. It achieves the highest overall F1-score with a value of 0.64. Precision remains competitive at 0.58. This pattern fits practical scenarios that require stable and reliable multi label predictions. The relative importance of precision and recall depends on the intended application. In exploratory peptide discovery and functional annotation, higher recall is generally desirable to minimize false negatives. In contrast, large-scale *in silico* screening may prioritize higher precision to reduce downstream experimental validation costs. Because SA-MTP outputs continuous confidence scores for each function, decision thresholds can be adjusted according to application requirements, enabling either high-recall screening or more conservative candidate prioritization.

### Ablation analysis

Under the same benchmarking protocol as TPpred-LE, we conducted ablation studies to quantify the contributions of key components in SA-MTP (Table 2 and Table 3).

**Table 2:** Macro-level performance comparison between PLM-only and graph-augmented variants

**Variant**	**MCC**	**F1**	**Precision**
PLM-only	0.391 $\pm $ 0.014	0.428 $\pm $ 0.014	0.494 $\pm $ 0.053
Graph-augmented	**0.399 $\pm $ 0.018**	**0.434 $\pm $ 0.021**	**0.515 $\pm $ 0.045**

**Table 3:** Macro-level performance comparison between shared-head and FiLM-enhanced graph-based variants

**Variant**	**MCC**	**F1**	**Precision**
Graph-augmented	0.399 $\pm $ 0.018	0.434 $\pm $ 0.021	0.515 $\pm $ 0.045
Graph + FiLM	**0.406 $\pm $ 0.028**	**0.441 $\pm $ 0.030**	**0.520 $\pm $ 0.047**

We first evaluated the impact of structure-aware graph modeling by comparing the graph-augmented variant with a PLM-only baseline that excludes dynamic structural modeling and graph attention. As shown in Table 2, introducing structure-aware graph encoding consistently improves macro-level MCC, F1-score, and precision. These results indicate that dynamic structural modeling enhances discriminative feature aggregation and improves prediction robustness under multi-label TP prediction settings. Detailed per-label ablation results are provided in Supplementary Methods Table E5.

We next assessed the contribution of the FiLM-based label-specific classification head by comparing the shared-head graph variant with its FiLM-enhanced counterpart. As summarized in Table 3, FiLM-based modulation further improves MCC, F1-score, and precision, suggesting enhanced label-specific representational capacity and more refined functional discrimination. The consistent improvements across composite metrics indicate that lightweight label-conditioned modulation can improve prediction quality without introducing instability into the graph-based encoder. Detailed per-label comparisons are provided in Supplementary Methods Table E6.

Comparisons with an embedding-based kNN graph baseline are provided in Supplementary Methods E3 and further support the effectiveness of the proposed structure-aware graph construction strategy.

Taken together, these ablation results demonstrate that structure-aware graph modeling and FiLM-based label modulation provide complementary benefits for multi-label TP function prediction. The integration of dynamic structural encoding with lightweight label-aware modulation enables SA-MTP to achieve more balanced, robust, and reliable performance across diverse TP functions.

### Threshold optimization

**Fig. 3.**
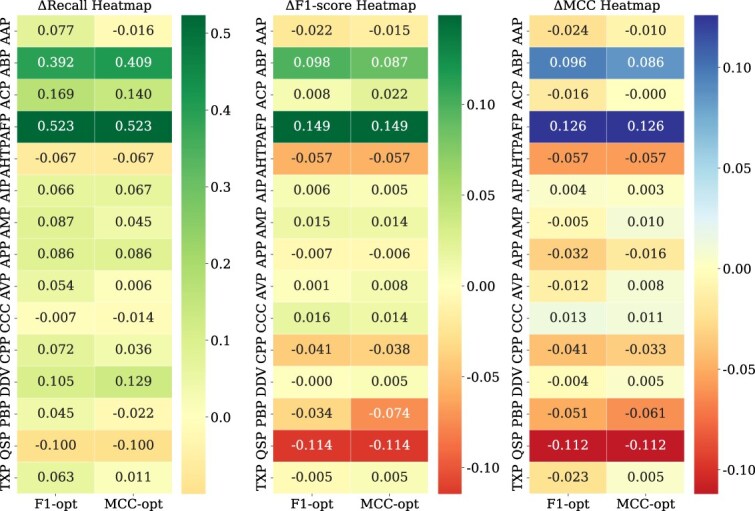
Performance comparison of optimization strategies.

To assess the impact of different threshold optimization strategies on multi-label TP function prediction, we systematically compared fixed thresholding with adaptive F1-optimized and MCC-optimized thresholding schemes. Changes in recall, F1-score, and MCC were evaluated relative to the fixed threshold baseline (Fig. 3).

Both adaptive thresholding strategies show performance improvement across multiple functional categories. The effect is clearer for labels with strong class imbalance. These results indicate that threshold optimization can partially mitigate decision bias induced by imbalanced label distributions, although the magnitude of improvement varies across metrics and functional classes.

The F1-optimized strategy consistently achieves larger gains in recall and F1-score, with especially pronounced recall improvements for sparsely represented or long-tail functional categories. In contrast, MCC-optimized thresholding produces more balanced improvements in overall discriminative performance, as reflected by consistently higher MCC values across several functional categories, while avoiding excessive increases in false positives. Some functional categories show low sensitivity to threshold changes. Performance varies only slightly under all three strategies. This result indicates that performance for these categories is mainly limited by inherent classification difficulty rather than threshold choice.

To determine whether the observed performance gains mainly originated from adaptive threshold optimization or from the proposed architecture itself, additional experiments were conducted using a conventional fixed threshold of 0.5. Under this setting, SA-MTP continued to outperform competing methods across most functional categories and evaluation metrics, indicating that the performance improvements primarily arose from the proposed structure-aware graph modeling and label-aware representation learning rather than threshold optimization alone. Detailed results are provided in Supplementary Methods E4.

### Hyperparameter sensitivity analysis

**Fig. 4.**
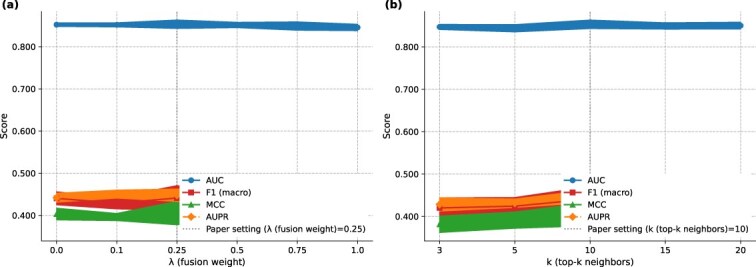
Sensitivity analysis of hyperparameters $\lambda $ and $k$.

We evaluated several key design choices in SA-MTP, including the relative contributions of SS2-derived structural similarity and ESM-2-derived contact priors, the graph fusion coefficient $\lambda $, and graph sparsification settings (Fig. 4).

**Fig. 5.**
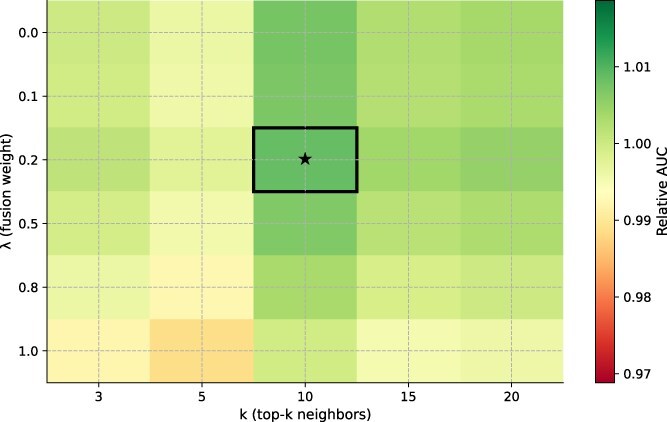
Heatmap of the effect of combined hyperparameter values on AUC performance.

To assess the contribution of each structural component, $\lambda $ was varied from 0 to 1. Specifically, $\lambda = 0$ corresponds to a graph constructed solely from SS2-derived structural similarity, whereas $\lambda = 1$ uses only ESM-derived contact priors. Both components contributed useful predictive information, while their integration achieved the best overall performance, indicating complementary structural contributions (Fig. 5).

Sensitivity analysis was further conducted using $\lambda \in \{0, 0.1, 0.25, 0.5,$  $0.75, 1.0\}$. Performance remained stable across the tested range, with macro-AUC varying by <1%. The best performance was obtained at $\lambda = 0.25$, indicating limited sensitivity to precise fusion parameter selection.

Graph sparsification was evaluated by varying the neighborhood size $k$ from 3 to 20. Performance differences were small across the tested range, although $k = 10$ produced the best overall results. These findings indicate that SA-MTP remains robust across different graph connectivity settings.

### Interpretability analysis

**Fig. 6.**
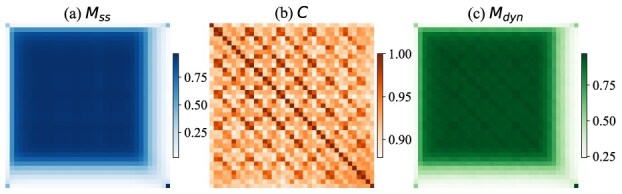
Dynamic structural graph construction for a representative $\alpha $-helical peptide sequence, with the three heatmaps showing (a) the secondary-structure-based similarity matrix $M_{ss}$, (b) the ESM-2-derived contact map $C$ capturing global semantic relationships, (c) the dynamically fused matrix $M_{dyn}$ integrating local structural and global contextual information.

**Fig. 7.**
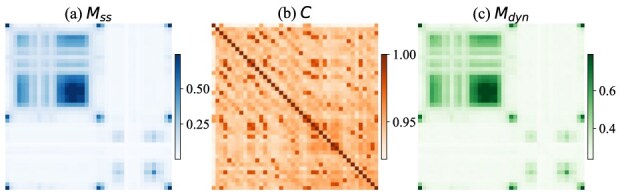
Dynamic structural graph construction for a representative mixed $\alpha $+$\beta $ peptide sequence, using the same visualization scheme as in Fig. 6.

**Fig. 8.**
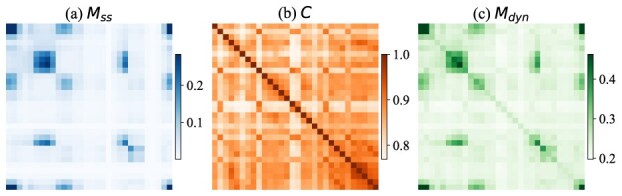
Dynamic structural graph construction for a representative high-entropy peptide sequence, using the same visualization scheme as in Fig. 6.

To qualitatively assess the effect of dynamic graph based structural modeling in combining local and global structure information, three representative peptide sequences were chosen from the test set. These sequences correspond to different levels of structural complexity. For each sequence, the secondary structure similarity matrix ($M_{\mathrm{ss}}$), the ESM-based contact map ($C$), and the dynamically fused adjacency matrix ($M_{\mathrm{dyn}}$) were visualized as three heatmaps in Figures (Figs 6– 8).

The selected sequences include regular $\alpha $-helical peptides, $\alpha $+$\beta $ mixed peptides with diverse local motifs, and high entropy peptides with strong conformational uncertainty. Figures 6–8 show different structure dependent connectivity patterns across these sequence types. In $\alpha $-helical sequences, $M_{\mathrm{ss}}$ displays smooth diagonal continuity. This reflects stable local structure. The same pattern remains in $M_{\mathrm{dyn}}$, with mild enhancement near structural boundaries. In $\alpha $+$\beta $ mixed sequences, $M_{\mathrm{dyn}}$ captures modular similarity patterns and long-range contact signals. These patterns reflect segment level structure and inter segment interactions. In high entropy sequences, secondary structure similarity appears diffuse. Contact signals remain weak. The resulting fused graphs are sparse and locally constrained, which limits the influence of unreliable structural information.

Together, these visual results show that dynamic graph construction adapts to sequence specific structural properties. It produces coherent connectivity for structurally regular peptides, modular patterns for mixed conformations, and sparse but stable representations under high uncertainty.

**Fig. 9.**
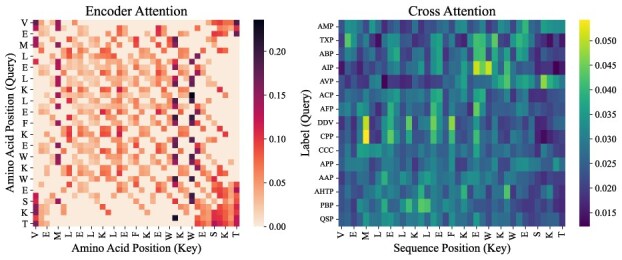
Attention visualization for a representative $\alpha $-helical peptide sequence. The left panel shows the normalized encoder self-attention (query versus key positions), while the right panel shows the normalized label-to-sequence cross-attention. Color intensity indicates the attention weight.

**Fig. 10.**
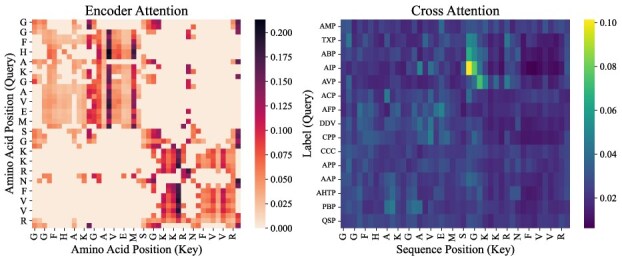
Attention visualization for a representative mixed $\alpha $+$\beta $ peptide sequence, using the same visualization scheme as Fig. 9.

**Fig. 11.**
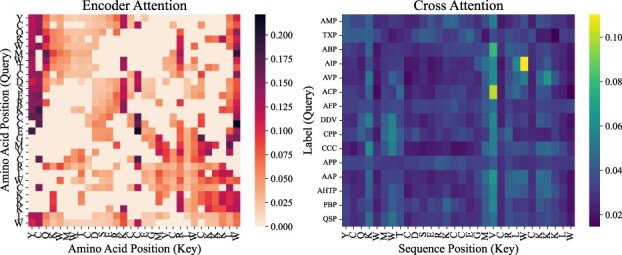
Attention visualization for a representative high-entropy peptide sequence, using the same visualization scheme as Fig. 9.

As shown in Figs 9–11, the encoder self-attention maps exhibit clear sequence-type-dependent aggregation behaviors. For structurally regular $\alpha $-helical peptides, attention patterns form smooth and coherent band-like structures along the sequence, indicating stable neighborhood aggregation within regular secondary-structure regions. In contrast, $\alpha $+$\beta $ mixed sequences display a combination of localized clustering and long-range attention across distinct structural segments, reflecting effective integration of local coherence and global contextual dependencies. For high entropy sequences, attention patterns appear fragmented and remain locally concentrated. This behavior reflects reliance on confident local regions when global structure is uncertain.

Cross-attention visualizations show label-specific focus patterns across different sequence types. For structurally regular peptides, label attention is concentrated and remains consistent within a small set of residue regions. For $\alpha $+$\beta $ mixed sequences, different functional labels attend to different structural modules. This pattern reflects distinct decision behaviors driven by heterogeneous structure. For high entropy sequences, cross attention maps still highlight localized high response regions for multiple labels. This shows that the model can extract useful residue level signals and preserve functional discrimination under noisy structural input.

These visual results indicate that the graph attention encoder adjusts neighborhood aggregation according to sequence-dependent structural complexity. The cross-attention mechanism integrates label semantics into sequence representations. Together, these behaviors allow SA-MTP to learn stable and interpretable attention patterns across structurally diverse peptides in multi-label prediction tasks.

To examine whether SA-MTP produces biologically meaningful predictions, a residue-level interpretability analysis was carried out on a representative antimicrobial peptide. This peptide was confidently predicted as an AMP with a score of 0.860. Integrated Gradients was used to measure residue wise contributions to the AMP prediction logit. Attention rollout was applied to the structure-aware GAT encoder to describe how structural information propagates through the dynamic graph. Together, these analyses reflect functional decision relevance and structure dependent information flow.

**Fig. 12.**
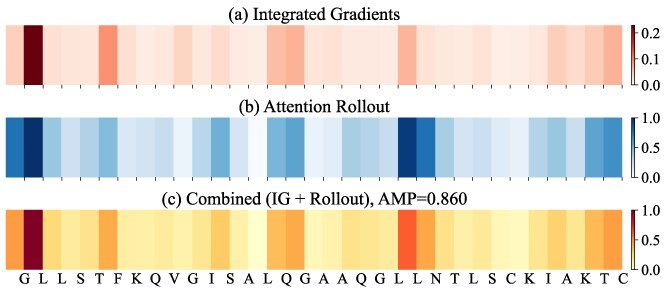
Residue-level interpretability analysis of peptide using Integrated Gradients and structure-aware attention rollout for AMP prediction.

As shown in Fig. 12, both IG attribution and attention rollout consistently emphasize a major hydrophobic cluster at the N terminus. This region plays an important role in AMP discrimination. Attribution signals also appear in the middle region and near the C terminus. These residues support helix stability and conformational strength. The observed patterns match common AMP characteristics. These include N terminal hydrophobic anchoring, amphipathic helix formation, and stabilization at peptide termini. The identified high-attention regions partially overlap with experimentally characterized functional regions reported for amphipathic antimicrobial peptides such as [50, 51], suggesting that SA-MTP captures biologically relevant residue patterns beyond global sequence similarity. However, the present analysis remains qualitative, and systematic benchmarking against experimentally resolved peptide–target complex structures would be required to rigorously evaluate attribution accuracy.

The peptide shows typical physicochemical features of membrane disruptive AMPs. These features include high hydrophobic residue content and a net positive charge. Consistency between IG attribution and structure-aware attention patterns indicates that SA-MTP captures residue regions associated with known AMP-related properties.

## Conclusion

The SA-MTP model combines sequence representations, probabilistic secondary-structure profiles, and dynamic graph-based structural modeling. This design captures the conformational flexibility commonly observed in TPs. Results show that SA-MTP outperforms existing methods in multi-label functional prediction, with especially notable recall improvements for long-tail functional categories. This improvement helps reduce false-negative predictions during early-stage peptide screening. SA-MTP models structure-aware residue interactions through dynamic graph construction and graph attention, enabling the framework to capture intrinsic conformational heterogeneity that is difficult to represent using sequence information alone. Consequently, the model supports more discriminative feature aggregation for highly flexible peptides and facilitates accurate and scalable high-throughput functional annotation. Although explicit 3D structure prediction methods such as AlphaFold2 and ESMFold may provide additional geometric information, TPs are often short, highly flexible, and partially disordered, which may limit the stability and reliability of single predicted conformations. In contrast, the probabilistic secondary-structure profiles and PLM-derived contact priors used in SA-MTP provide lightweight and uncertainty-aware structural representations that are more compatible with the conformational heterogeneity commonly observed in TPs. This design also enables computationally efficient large-scale peptide screening.

**Key Points**

- Multifunctional therapeutic peptides exhibit pronounced conformational heterogeneity, which limits the effectiveness of sequence-only prediction models.
- We propose SA-MTP, a structure-aware multi-label framework that integrates pretrained protein language models with dynamic graph-based structural modeling.
- By explicitly modeling probabilistic secondary-structure similarity and residue–residue contact priors, SA-MTP captures flexible and input-dependent peptide conformations.
- Benchmark experiments show that SA-MTP achieves superior overall performance compared with existing methods, with notable advantages for imbalanced and long-tail functional categories.
- Structure-aware attention and attribution analyses highlight residue-level patterns associated with therapeutic peptide activities.

## Supplementary Material

Supplementary_Methods_bbag361

## Data Availability

The source code and dataset are available on https://github.com/LZW-TECH/SA-MTP.

## References

[1] Rosson E, François Lux L, David YG et al. Focus on therapeutic peptides and their delivery. International Journal of Pharmaceutics 2025;675:125555. 10.1016/j.ijpharm.2025.125555 40194730

[2] Wang L, Wang N, Zhang W et al. Therapeutic peptides: current applications and future directions. *Signal Transduct Target Ther* 2022;7:48. 35165272 10.1038/s41392-022-00904-4 PMC8844085

[3] Sharma D, Dhiman I, Das S et al. Recent advances in therapeutic peptides: innovations and applications in treating infections and diseases. *ACS Omega* 2025;10:17087–107. 40352490 10.1021/acsomega.5c02077 PMC12059905

[4] Liscano Y, Oñate-Garzón J, Delgado JP. Peptides with dual antimicrobial–anticancer activity: strategies to overcome peptide limitations and rational design of anticancer peptides. *Molecules* 2020;25:4245. 32947811 10.3390/molecules25184245 PMC7570524

[5] Makhoba XH, Jr CV, Mosa RA et al. Potential impact of the multi-target drug approach in the treatment of some complex diseases. Drug Design, Development and Therapy 2020;14:3235–49. 10.2147/DDDT.S257494 PMC7440888 32884235

[6] Song Z, Li H, Ge F et al. METFAN: multisource enhanced therapeutic peptide function prediction via adapter network. ACS omega 2025;10:53019–38. 10.1021/acsomega.5c07552 PMC12622265 41255570

[7] Li T, Fan H, Zhao J et al. MultiPep-DLCL: recognition of multifunctional therapeutic peptides through deep learning with label-sequence contrastive learning. *Brief Bioinform* 2025;26:bbaf274. 40518951 10.1093/bib/bbaf274 PMC12167766

[8] Wang G, Li X, Wang Z. APD3: the antimicrobial peptide database as a tool for research and education. *Nucleic Acids Res* 2016; 44:D1087–93. 26602694 10.1093/nar/gkv1278 PMC4702905

[9] Jhong J-H, Chi Y-H, Li W-C et al. dbAMP: an integrated resource for exploring antimicrobial peptides with functional activities and physicochemical properties on transcriptome and proteome data. *Nucleic Acids Res* 2019;47:D285–97. 30380085 10.1093/nar/gky1030 PMC6323920

[10] Kang X, Dong F, Shi C et al. DRAMP 2.0, an updated data repository of antimicrobial peptides. *Sci Data* 2019;6:148. 31409791 10.1038/s41597-019-0154-y PMC6692298

[11] Wei L, Zhou C, Ran S et al. PEPred-Suite: improved and robust prediction of therapeutic peptides using adaptive feature representation learning. *Bioinformatics* 2019;35:4272–80. 30994882 10.1093/bioinformatics/btz246

[12] Zhang YP, Zou Q. PPTPP: a novel therapeutic peptide prediction method using physicochemical property encoding and adaptive feature representation learning. *Bioinformatics* 2020; 36:3982–7. 32348463 10.1093/bioinformatics/btaa275

[13] Tyagi A, Kapoor P, Kumar R et al. In silico models for designing and discovering novel anticancer peptides. *Sci Rep* 2013;3:2984. 24136089 10.1038/srep02984 PMC6505669

[14] Chen W, Ding H, Feng P et al. iACP: a sequence-based tool for identifying anticancer peptides. *Oncotarget* 2016;7:16895. 26942877 10.18632/oncotarget.7815 PMC4941358

[15] Li F-M, Wang X-Q. Identifying anticancer peptides by using improved hybrid compositions. *Sci Rep* 2016;6:33910. 27670968 10.1038/srep33910 PMC5037382

[16] Lin W, Dong X. Imbalanced multi-label learning for identifying antimicrobial peptides and their functional types. *Bioinformatics* 2016;32:3745–52. 27565585 10.1093/bioinformatics/btw560 PMC5167070

[17] Lezheng Y, Jing R, Liu F et al. DeepACP: a novel computational approach for accurate identification of anticancer peptides by deep learning algorithm. *Mol Ther Nucleic Acids* 2020;22:862–70. 33230481 10.1016/j.omtn.2020.10.005 PMC7658571

[18] Ahmed S, Muhammod R, Khan ZH et al. ACP-MHCNN: an accurate multi-headed deep-convolutional neural network to predict anticancer peptides. *Sci Rep* 2021;11:23676. 34880291 10.1038/s41598-021-02703-3 PMC8654959

[19] Chen J, Cheong HH, Siu SWI. xDeep-AcPEP: deep learning method for anticancer peptide activity prediction based on convolutional neural network and multitask learning. *J Chem Inf Model* 2021;61:3789–803. 34327990 10.1021/acs.jcim.1c00181

[20] Grønning AGB, Kacprowski T, Scheele C. MultiPep: a hierarchical deep learning approach for multi-label classification of peptide bioactivities. *Biol Methods Protoc* 2021;6:bpab021. 34909478 10.1093/biomethods/bpab021 PMC8665375

[21] Tang W, Dai R, Yan W et al. Identifying multi-functional bioactive peptide functions using multi-label deep learning. Briefings in bioinformatics 2022;23:bbab414. 10.1093/bib/bbab414 34651655

[22] Jing X, Li F, Li C et al. iAMPCN: a deep-learning approach for identifying antimicrobial peptides and their functional activities. *Brief Bioinform* 2023;24:bbad240. 37369638 10.1093/bib/bbad240 PMC10359087

[23] Ge F, Zhou J, Zhang M et al. MFP-MFL: leveraging graph attention and multi-feature integration for superior multifunctional bioactive peptide prediction. *Int J Mol Sci* 2025;26:1317. 39941085 10.3390/ijms26031317 PMC11818429

[24] Dee W . LMPred: predicting antimicrobial peptides using pre-trained language models and deep learning. *Bioinf Adv* 2022;2:vbac021. 10.1093/bioadv/vbac021 PMC9710646 36699381

[25] Guntuboina C, Das A, Mollaei P et al. PeptideBERT: a language model based on transformers for peptide property prediction. *J Phys Chem Lett* 2023;14:10427–34. 37956397 10.1021/acs.jpclett.3c02398 PMC10683064

[26] Yan K, Lv H, Guo Y et al. TPpred-ATMV: therapeutic peptide prediction by adaptive multi-view tensor learning model. *Bioinformatics* 2022;38:2712–8. 35561206 10.1093/bioinformatics/btac200

[27] Lv H, Yan K, Liu B. TPpred-LE: therapeutic peptide function prediction based on label embedding. *BMC Biol* 2023;21:238. 37904157 10.1186/s12915-023-01740-w PMC10617231

[28] Yan K, Lv H, Shao J et al. TPpred-SC: multi-functional therapeutic peptide prediction based on multi-label supervised contrastive learning. *Sci China Inf Sci* 2024;67:212105.

[29] Tuo S, Zhu YL, Lin J et al. AMHF-TP: multifunctional therapeutic peptides prediction based on multi-granularity hierarchical features. *Quant Biol* 2025;13:e73. 41675372 10.1002/qub2.73 PMC12806036

[30] Nguyen LT, Haney EF, Vogel HJ. The expanding scope of antimicrobial peptide structures and their modes of action. *Trends Biotechnol* 2011;29:464–72. 21680034 10.1016/j.tibtech.2011.05.001

[31] Li X, Li Y, Han H et al. Solution structures of human LL-37 fragments and NMR-based identification of a minimal membrane-targeting antimicrobial and anticancer region. *J Am Chem Soc* 2006;128:5776–85. 16637646 10.1021/ja0584875

[32] Voronko OE, Khotina VA, Kashirskikh DA et al. Antimicrobial peptides of the cathelicidin family: focus on LL-37 and its modifications. *Int J Mol Sci* 2025;26:8103. 40869425 10.3390/ijms26168103 PMC12386566

[33] Latendorf T, Gerstel U, Zhihong W et al. Cationic intrinsically disordered antimicrobial peptides (CIDAMPs) represent a new paradigm of innate defense with a potential for novel anti-infectives. *Sci Rep* 2019;9:3331. 30833614 10.1038/s41598-019-39219-w PMC6399351

[34] Jain S, Wallace BC. Attention is not explanation. Proceedings of the 2019 Conference of the North American Chapter of the Association for Computational Linguistics: Human Language Technologies, Volume 1 (Long and Short Papers). 2019, 3543–56.

[35] Serrano S, Smith NA. Is attention interpretable? Proceedings of the 57th Annual Meeting of the Association for Computational Linguistics. 2019, 2931–51.

[36] Rao R, Meier J, Sercu T et al. Transformer protein language models are unsupervised structure learners. International Conference on Learning Representations (ICLR) 2021.

[37] Buda M, Maki A, Mazurowski MA. A systematic study of the class imbalance problem in convolutional neural networks. *Neural Netw* 2018;106:249–59. 30092410 10.1016/j.neunet.2018.07.011

[38] McGuffin LJ, Bryson K, Jones DT. The psipred protein structure prediction server. *Bioinformatics* 2000;16:404–5. 10869041 10.1093/bioinformatics/16.4.404

[39] Lin Z, Akin H, Rao R et al. Evolutionary-scale prediction of atomic-level protein structure with a language model. *Science* 2023;379:1123–30. 36927031 10.1126/science.ade2574

[40] Rives A, Meier J, Sercu T et al. Biological structure and function emerge from scaling unsupervised learning to 250 million protein sequences. *Proc Natl Acad Sci* 2021;118:e2016239118. 33876751 10.1073/pnas.2016239118 PMC8053943

[41] Fantini J, Azzaz F, Di Scala C et al. Conformationally adaptive therapeutic peptides for diseases caused by intrinsically disordered proteins (IDPs). New paradigm for drug discovery: target the target, not the arrow. Pharmacology & Therapeutics 2025;267:108797. 10.1016/j.pharmthera.2025.108797 39828029

[42] Veličković P, Cucurull G, Casanova A et al. Graph attention networks. International Conference on Learning Representations (ICLR) 2018.

[43] Lai B, Jinbo X. Accurate protein function prediction via graph attention networks with predicted structure information. *Brief Bioinform* 2022;23:bbab502. 34882195 10.1093/bib/bbab502 PMC8898000

[44] Draszawka K, Szymański J. From scores to predictions in multi-label classification: neural thresholding strategies. *Appl Sci* 2023; 13:7591.

[45] Yang L, Wu X-Z, Jiang Y et al. Multi-label learning with deep forest. Frontiers in Artificial Intelligence and Applications 2020;325:1634–41.

[46] Yan W, Tang W, Wang L et al. PrMFTP: multi-functional therapeutic peptides prediction based on multi-head self-attention mechanism and class weight optimization. *PLoS Comput Biol* 2022; 18:e1010511. 36094961 10.1371/journal.pcbi.1010511 PMC9499272

[47] Li Y, Li X, Liu Y et al. MPMABP: a CNN and Bi-LSTM-based method for predicting multi-activities of bioactive peptides. *Pharmaceuticals* 2022;15:707. 35745625 10.3390/ph15060707 PMC9231127

[48] Otovic E, Njirjak M, Kalafatovic D et al. Sequential properties representation scheme for recurrent neural network-based prediction of therapeutic peptides. *J Chem Inf Model* 2022;62:2961–72. 35704881 10.1021/acs.jcim.2c00526

[49] Fan H, Yan W, Wang L et al. Deep learning-based multi-functional therapeutic peptides prediction with a multi-label focal dice loss function. *Bioinformatics* 2023;39:btad334. 37216900 10.1093/bioinformatics/btad334 PMC10234765

[50] Ding B, Soblosky L, Nguyen K et al. Physiologically-relevant modes of membrane interactions by the human antimicrobial peptide, LL-37, revealed by SFG experiments. *Sci Rep* 2013;3:1854. 23676762 10.1038/srep01854 PMC3655398

[51] Marquette A, Bechinger B. Biophysical investigations elucidating the mechanisms of action of antimicrobial peptides and their synergism. *Biomolecules* 2018;8:18. 29670065 10.3390/biom8020018 PMC6023007

