# Supplementary material for: SA-MTP: a structure-aware framework for multifunctional therapeutic peptide annotation
Source: Brief Bioinform. 2026 Jul 3;27(4):bbag361. doi: 10.1093/bib/bbag361 (PMC13331450; doi:10.1093/bib/bbag361)
Supplement: Supplementary_Methods_bbag361 [file supplementary_methods_bbag361.pdf]

Supplementary Methods

S1 SS2 Generation and Embedding Preprocessing

For workflow implementation, each peptide sequence is stored in standard FASTA format, and SS2 files are generated automatically using two PSIPRED modes. The runpsipredplus mode leverages PSI-BLAST searches against the UniRef50 database to construct a position-specific scoring matrix (PSSM), enabling high-accuracy secondary-structure prediction. For short peptides lacking detectable homologous sequences, the runpsipred_single mode is employed for single-sequence prediction. The entire pipeline is executed using parallelized scripts to ensure computational efficiency and complete dataset coverage. Ultimately, each peptide is associated with a normalized SS2 probability matrix $\mathbf{S} \in \mathbb{R}^{L\times 3}$, where L denotes the peptide length.

S2 Residue-Level Sequence Representation and Projection

Given a peptide sequence $S = (a_1, a_2, \dots, a_L)$ of length L,the pretrained protein language model ESM-2 produces residue-level contextual embeddings:

$$H_{\text{esm}} = \left[h_1^{\text{esm}}, h_2^{\text{esm}}, \dots, h_L^{\text{esm}}\right]^\top \in \mathbb{R}^{L\times 1280}, \tag{1}$$

where $h_i^{\text{esm}} \in \mathbb{R}^{1280}$ denotes the embedding of residue i.

To reduce dimensionality and align feature representations with the downstream graph encoder, a two-layer projection module is applied.

First projection layer (1280 → 512)

$$\begin{aligned} H_1 &= \text{LayerNorm}(H_{\text{esm}} W_1 + b_1), \\ H_1 &= \text{GELU}(H_1), \\ H_1 &= \text{Dropout}(H_1), \end{aligned} \tag{2}$$

where $W_1 \in \mathbb{R}^{1280\times 512}$ and $b_1 \in \mathbb{R}^{512}$.

Second projection layer (512 → 256)

$$\begin{aligned} H_{\text{plm}} &= \text{LayerNorm}(H_1 W_2 + b_2), \\ H_{\text{plm}} &= \text{Dropout}(H_{\text{plm}}), \end{aligned} \tag{3}$$

where $W_2 \in \mathbb{R}^{512\times 256}$ and $b_2 \in \mathbb{R}^{256}$.

The resulting matrix $H_{\text{plm}} \in \mathbb{R}^{L\times 256}$ serves as the residue-level sequence-semantic representation for subsequent structure-aware graph encoding.

S3 Dynamic Structure-Aware Graph Construction

Structural similarity computation

From PSIPRED SS2 outputs, each residue i is associated with a secondary-structure probability vector $S_i = [P_H(i), P_E(i), P_C(i)]$, where $P_H + P_E + P_C = 1$.

For residues i and j, secondary-structure dissimilarity is quantified using the Jensen–Shannon divergence (JSD). The intermediate distribution is defined as

$$M_{ij} = \frac{1}{2}(S_i + S_j). \tag{4}$$

The JSD is computed as

$$D_{\mathrm{JS}}(S_i, S_j) = \frac{1}{2}D_{\mathrm{KL}}(S_i \parallel M_{ij}) + \frac{1}{2}D_{\mathrm{KL}}(S_j \parallel M_{ij}), \tag{5}$$

where

$$D_{\mathrm{KL}}(S_i \parallel M_{ij}) = \sum_{k\in\{H,E,C\}} P_k(i) \log \frac{P_k(i)}{M_k(i,j)}. \tag{6}$$

Structural similarity is obtained via an exponential kernel:

$$M_{\mathrm{ss}}[i,j] = \exp\left(-\beta D_{\mathrm{JS}}(S_i, S_j)\right), \tag{7}$$

where $\beta = 4.0$.

Entropy-based structural confidence weighting

Residue-level structural uncertainty is quantified by entropy:

$$H(S_i) = -\sum_{k\in\{H,E,C\}} P_k(i) \log_2 P_k(i). \tag{8}$$

Entropy is normalized to obtain a confidence score

$$\begin{aligned} c_i &= 1 - \frac{\log_2 3}{H(S_i)}, \\ c_i &\in [0,1]. \end{aligned} \tag{9}$$

The confidence-weighted structural similarity matrix is defined as

$$M_{\mathrm{ss}}^{\mathrm{conf}}[i,j] = c_i \cdot c_j \cdot M_{\mathrm{ss}}[i,j], \tag{10}$$

or equivalently,

$$M_{\mathrm{ss}} \leftarrow \mathrm{diag}(c) \cdot M_{\mathrm{ss}} \cdot \mathrm{diag}(c). \tag{11}$$

Incorporation of contact information

Residue–residue contact probabilities predicted by ESM-2 are denoted by $C \in [0,1]^{L\times L}$.

Local structural similarity and long-range contact priors are fused as

$$M_{\mathrm{dyn}} = (1-\lambda)M_{\mathrm{ss}}^{\mathrm{conf}} + \lambda C, \tag{12}$$

where $\lambda = 0.25$.

Sparsification, symmetrization, and DropEdge regularization

To sparsify the graph, only the top-k neighbors per residue are retained:

$$k = \min(10, L - 1). \tag{13}$$

The sparsified matrix is defined as

$$M_{sp}[i, j] = \begin{cases} M_{\text{dyn}}[i, j], & j \in N_k(i), \\ 0, & \text{otherwise.} \end{cases} \tag{14}$$

Symmetrization yields the final adjacency matrix

$$M = \frac{1}{2}(M_{sp} + M_{sp}^{\top}), \tag{15}$$

which is used as input to the GAT encoder.

During training, DropEdge regularization is applied:

$$\widetilde{M}[i, j] = \begin{cases} 0, & \text{with probability } p_{\text{drop}}, \\ M[i, j], & \text{with probability } 1 - p_{\text{drop}}, \end{cases} \tag{16}$$

with $p_{drop} = 0.1$. During inference, the full matrix M is used.

S4 Structure-Aware Graph Attention Encoding

Given the projected residue feature matrix $H_{\text{plm}} \in \mathbb{R}^{L \times 256}$ and the dynamic weighted adjacency matrix $M \in \mathbb{R}^{L \times L}$, each residue is treated as a graph node with initial feature $h_i^{(0)} = h_{\text{plm},i}$. The adjacency matrix M defines structure-aware neighborhood constraints for graph attention–based message passing.

Single-head graph attention

Let the input to the l-th GAT layer be $H^{(l)} = [h_1^{(l)}, \ldots, h_L^{(l)}]^{\top} \in \mathbb{R}^{L \times d_l}$.

A linear transformation is applied:

$$z_i^{(l)} = W^{(l)} h_i^{(l)}, \\ W^{(l)} \in \mathbb{R}^{d_l \times d_{l+1}}. \tag{17}$$

For any permissible edge (i, j) such that $M[i, j] > 0$, the unnormalized attention score is

$$e_{ij}^{(l)} = \text{LeakyReLU}\left(a^{(l)\top}\left[z_i^{(l)} \parallel z_j^{(l)}\right]\right), \tag{18}$$

where $a^{(l)} \in \mathbb{R}^{2d_{l+1}}$.

Let $N(i) = \{j \mid M[i, j] > 0\}$ denote the structure-aware neighborhood of node i.

Normalized attention coefficients are computed as

$$\alpha_{ij}^{(l)} = \frac{\exp(e_{ij}^{(l)})}{\sum_{k\in N(i)} \exp(e_{ik}^{(l)})}, \quad j \in N(i). \tag{19}$$

Node representations are updated via

$$h_i^{(l+1)} = \sigma\left(\sum_{j\in N(i)} \alpha_{ij}^{(l)} z_j^{(l)}\right), \tag{20}$$

where σ denotes the GELU activation.

In matrix form,

$$\begin{aligned} H^{(l+1)} &= \sigma\left(A^{(l)} Z^{(l)}\right), \\ Z^{(l)} &= H^{(l)} W^{(l)}, \end{aligned} \tag{21}$$

with

$$\alpha_{ij}^{(l)} = 0 \iff M[i,j] = 0. \tag{22}$$

Multi-head graph attention

Each GAT layer employs $K = 4$ attention heads. For head k, independent parameters $W^{(l,k)}$ and $a^{(l,k)}$ are used, producing $h_i^{(l+1,k)}$.

The multi-head output is obtained by concatenation:

$$h_i^{(l+1)} = \sum_{k=1}^{K} h_i^{(l+1,k)}, \tag{23}$$

or equivalently,

$$H^{(l+1)} = \left[H^{(l+1,1)} \parallel H^{(l+1,2)} \parallel \cdots \parallel H^{(l+1,K)}\right]. \tag{24}$$

Each head outputs $\frac{d_{l+1}}{K}$ features, preserving the total dimensionality.

Residual connections and regularization

When input and output dimensions match, residual connections are applied:

$$H^{(l+1)} \leftarrow H^{(l+1)} + H^{(l)}. \tag{25}$$

Dropout is applied to feature outputs:

$$\widetilde{H}^{(l+1)} = \text{Dropout}\left(H^{(l+1)}\right). \tag{26}$$

Layer normalization and GELU activation are applied after aggregation.

Output representation

After two stacked multi-head GAT layers, the model produces the final structure-aware residue representation $H_{\text{gat}} \in \mathbb{R}^{L\times 256}$, which is used as input to the label-aware FiLM-based classification module.

S5 Label-Aware Cross-Attention and FiLM-Based Modulation

Label embedding

Given $C = 15$ therapeutic function labels, a learnable label embedding matrix is defined as

$$E_{\text{label}} = \begin{bmatrix} e_1^\top \\ e_2^\top \\ \vdots \\ e_C^\top \end{bmatrix} \in \mathbb{R}^{C\times 256}, \tag{27}$$

where each $e_i \in R^{256}$ denotes the embedding of label i.

Cross-attention for sequence–label interaction

Let $H_{\text{gat}} \in \mathbb{R}^{L\times 256}$ denote the structure-aware residue representations. Cross-attention is computed with label embeddings as queries and residue representations as keys and values:

$$\begin{aligned} Q &= E_{\text{label}} W_Q, \\ K &= H_{\text{gat}} W_K, \\ V &= H_{\text{gat}} W_V, \end{aligned} \tag{28}$$

where $W_Q, W_K, W_V \in \mathbb{R}^{256\times 256}$.

For label i, attention weights over residues are

$$\begin{aligned} \alpha_i &= \text{softmax}\left(\frac{Q_i K^\top}{\sqrt{d}}\right), \\ d &= 256, \end{aligned} \tag{29}$$

yielding the attended representation

$$z_i = \alpha_i V \in \mathbb{R}^{1\times 256}. \tag{30}$$

Stacking all labels produces

$$Z = \text{Attention}(E_{\text{label}}, H_{\text{gat}}, H_{\text{gat}}) \in \mathbb{R}^{C\times 256}. \tag{31}$$

FiLM-based label-specific modulation

For label i, a modulation vector is generated as

$$\begin{aligned} s_i &= \sigma(W_s e_i), \\ W_s &\in \mathbb{R}^{256\times 256}, \end{aligned} \tag{32}$$

where $\sigma(\cdot)$ denotes the sigmoid function.

Feature-wise linear modulation is applied using scaling only:

$$z_i = (1 + \alpha \cdot s_i) \odot z_i, \alpha = 0.2, \tag{33}$$

with $\odot$ denoting element-wise multiplication. Parameters in W_s are initialized to zero.

Shared classification head and final prediction

Each modulated label representation is passed through a shared linear classifier:

$$y_i = \sigma(w^\top z_i + b), \tag{34}$$

producing the final prediction vector

$$y = [y_1, y_2, \ldots, y_C] \in [0, 1]^C. \tag{35}$$

E1 Distribution of therapeutic peptide categories across dataset partitions

The TPpred-LE benchmark dataset was generated after redundancy reduction using CD-HIT at a 90% sequence identity threshold and subsequently divided into training, validation, and independent test subsets using an 8:1:1 split. To provide additional transparency regarding the benchmark partition adopted in this study, Table E1 summarizes the numbers and proportions of all 15 therapeutic peptide categories in the three subsets.

As shown in Table E1, all therapeutic peptide categories are represented in the training, validation, and test sets. Although some variation is observed for several low-frequency categories, the overall label distributions remain broadly comparable across the three subsets.

Table E1. Distribution of therapeutic peptide categories in the training, validation, and test subsets.

Function	Training	Validation	Test
AMP	3,587 (43.80%)	501 (48.97%)	500 (48.83%)
TXP	1,865 (22.77%)	250 (24.44%)	230 (22.46%)
ABP	1,355 (16.54%)	242 (23.66%)	150 (14.65%)
AIP	1,367 (16.69%)	199 (19.45%)	149 (14.55%)
AVP	1,204 (14.70%)	150 (14.66%)	139 (13.57%)
ACP	776 (9.47%)	110 (10.75%)	83 (8.11%)
AFP	647 (7.90%)	115 (11.24%)	73 (7.13%)
DDV	454 (5.54%)	57 (5.57%)	59 (5.76%)
CPP	450 (5.49%)	56 (5.47%)	50 (4.88%)
CCC	227 (2.77%)	28 (2.74%)	29 (2.83%)
APP	114 (1.39%)	23 (2.25%)	14 (1.37%)
AAP	107 (1.31%)	15 (1.47%)	13 (1.27%)
AHTP	78 (0.95%)	9 (0.88%)	6 (0.59%)
PBP	52 (0.63%)	7 (0.68%)	9 (0.88%)
QSP	40 (0.49%)	7 (0.68%)	6 (0.59%)

E2 Generalization under low-homology conditions

To further evaluate the generalization capability of SA-MTP on previously unseen peptide sequences, we constructed an additional low-homology benchmark using a stricter sequence identity threshold. Specifically, CD-HIT was applied with a 40% sequence identity cutoff prior to dataset partitioning,

substantially reducing homologous overlap between training and test samples relative to the standard benchmark protocol inherited from TPpred-LE.

Under this substantially more challenging setting, SA-MTP achieved a macro-AUC of 0.738 ± 0.011 across five independent runs, while maintaining a macro-AUPR of 0.304 ± 0.025 and an overall accuracy of 0.907 ± 0.004. In addition, 9 out of 15 therapeutic peptide categories maintained AUC values above 0.70, including several function classes such as AIP, PBP, TXP, and CCC.

Although performance decreased relative to the standard benchmark setting, the model retained meaningful discriminative capability under strict homology constraints. These results suggest that SA-MTP captures transferable sequence–structure–function relationships beyond simple sequence similarity memorization, while also highlighting the increased difficulty of multi-label therapeutic peptide prediction under low-homology conditions.

We note that standardized external multi-label therapeutic peptide benchmarks remain limited in the current literature. Future work may therefore investigate broader cross-dataset validation and prospective evaluation on newly released peptide databases as such resources become available.

Table E2. Performance of SA-MTP under standard and low-homology benchmark settings.

Setting	Macro-AUC	Macro-AUPR	ACC
Standard benchmark (90%)	0.853	0.448	0.926
Low-homology benchmark (40%)	0.738 ± 0.011	0.304 ± 0.025	0.907 ± 0.004

E3 Comparison with embedding-based kNN graphs

To further evaluate the contribution of the proposed structure-aware graph construction strategy, we compared SA-MTP with an embedding-based kNN graph baseline in which graph edges were constructed solely using k-nearest neighbors in the projected ESM-2 residue embedding space, without incorporating SS2-derived structural similarity or ESM-derived contact priors.

Under the same training and evaluation settings, the proposed structure-aware graph consistently achieved improved performance across macro-level metrics, including macro-AUC, macro-AUPR, macro-F1, and macro-MCC. The improvements remained stable across multiple evaluation runs.

These results suggest that probabilistic secondary-structure similarity and contact-aware priors provide complementary relational information beyond embedding proximity alone. At the same time, the relatively strong performance of the embedding-based kNN graph indicates that pretrained residue embeddings already encode substantial biochemical, evolutionary, and contextual information. Together, these findings support the effectiveness of the proposed structure-aware graph construction strategy while further highlighting the representational capability of modern protein language models.

Table E3. Performance comparison between embedding-based kNN and proposed dynamic graph construction.

Graph construction	Macro-AUC	Macro-AUPR	Macro-F1	Macro-MCC
Embedding-based kNN	0.846 ± 0.007	0.435 ± 0.007	0.418 ± 0.011	0.381 ± 0.011
Proposed dynamic graph	0.853	0.448	0.441	0.406

E4 Fixed-threshold evaluation analysis

Most baseline methods reported in previous studies either used a conventional fixed threshold of 0.5 or did not explicitly describe threshold optimization procedures in sufficient detail for exact reproduction. To determine whether the performance gains of SA-MTP primarily arise from adaptive threshold optimization, we additionally evaluated SA-MTP using the same fixed-threshold setting (0.5) under an identical evaluation protocol. Under this setting, SA-MTP continued to outperform competing approaches across the major evaluation metrics, indicating that the observed improvements are not solely attributable to adaptive threshold selection but primarily arise from the proposed structure-aware graph modeling and label-aware representation learning framework.

Table E4. Performance comparison under fixed-threshold evaluation.

Function	Method	AUC	MCC	F1
AAP	PEPred-Suite[a]	0.577	0.02	0.03
	PPTPP[ab]	0.604	0.037	0.033
	TPpred-ATMV[a]	0.583	0.009	0.027
	TPpred-LE	0.745	0.278	0.285
	SA-MTP	**0.807±0.026**	**0.374±0.096**	**0.361±0.090**
ABP	PEPred-Suite[a]	0.744	0.261	0.367
	PPTPP[ab]	0.732	0.261	0.365
	TPpred-ATMV[a]	0.731	0.256	0.36
	TPpred-LE	0.834	0.337	0.426
	SA-MTP	**0.843±0.016**	**0.347±0.054**	0.419±0.080
AIP	PEPred-Suite[a]	0.363	-0.19	0.18
	PPTPP[ab]	0.386	-0.06	0.168
	TPpred-ATMV[a]	0.369	-0.25	0.196
	TPpred-LE	0.895	0.527	0.594
	SA-MTP	**0.920±0.009**	**0.584±0.014**	**0.644±0.013**
AVP	PEPred-Suite[a]	0.382	-0.129	0.147
	PPTPP[ab]	0.404	-0.11	0.169
	TPpred-ATMV[a]	0.394	-0.118	0.135
	TPpred-LE	0.835	0.457	0.529
	SA-MTP	**0.847±0.005**	**0.500±0.029**	**0.556±0.014**
CPP	PEPred-Suite[a]	0.813	0.152	0.142
	PPTPP[ab]	0.814	0.14	0.139
	TPpred-ATMV[a]	0.815	0.152	0.139
	TPpred-LE	0.899	0.477	0.502
	SA-MTP	**0.910±0.013**	**0.526±0.043**	**0.546±0.043**
PBP	PEPred-Suite[a]	0.907	0.153	0.069
	PPTPP[ab]	0.829	0.119	0.07
	TPpred-ATMV[a]	0.836	0.153	0.086
	TPpred-LE	0.934	0.443	0.430
	SA-MTP	**0.964±0.01**	**0.517±0.134**	**0.492±0.119**
QSP	PEPred-Suite[a]	0.835	0.113	0.043
	PPTPP[ab]	0.815	0.08	0.033
	TPpred-ATMV[a]	0.772	0.054	0.027
	TPpred-LE	0.879	0.420	0.391
	SA-MTP	**0.955±0.033**	**0.467±0.170**	**0.448±0.166**

All methods were evaluated using a fixed decision threshold to assess whether the performance gains of SA-MTP primarily arise from adaptive threshold optimization.

Table E5. Performance comparison of PLM-only and graph-augmented variants

Function	Method	BACC	MCC	F1
ABP	SA-MTP	0.645	0.330	0.398
	SA-MTP[a]	**0.646**	**0.333**	**0.399**
AFP	SA-MTP	0.548	0.154	0.162
	SA-MTP[a]	**0.555**	**0.17**	**0.173**
AHTP	SA-MTP	0.532	0.102	0.079
	SA-MTP[a]	**0.549**	**0.118**	**0.114**
AIP	SA-MTP	0.795	0.562	0.624
	SA-MTP[a]	**0.796**	**0.577**	**0.636**
APP	SA-MTP	0.499	-0.005	0.000
	SA-MTP[a]	**0.520**	**0.069**	**0.061**
CCC	SA-MTP	0.791	0.591	0.593
	SA-MTP[a]	**0.798**	**0.606**	**0.613**
DDV	SA-MTP	0.678	0.397	0.420
	SA-MTP[a]	**0.688**	**0.415**	**0.440**
QSP	SA-MTP	0.716	0.538	0.509
	SA-MTP[a]	**0.7164**	**0.599**	**0.551**
TXP	SA-MTP	0.794	0.622	0.696
	SA-MTP[a]	**0.7944**	**0.623**	**0.698**

[a] The model uses dynamic graphs and GAT.

Table E6. Performance comparison between shared-head and FiLM-enhanced graph-based variants

Function	Method	BACC	MCC	AUPR	F1
AAP	SA-MTP[a]	0.643	0.327	0.176	0.323
	SA-MTP[ab]	0.637	**0.374**	**0.182**	**0.361**
ABP	SA-MTP[a]	0.646	0.333	0.443	0.399
	SA-MTP[ab]	**0.662**	**0.347**	**0.444**	**0.419**
ACP	SA-MTP[a]	0.619	0.305	0.361	0.324
	SA-MTP[ab]	**0.63**5	**0.323**	**0.37**	**0.358**
AFP	SA-MTP[a]	0.555	0.170	0.252	0.173
	SA-MTP[ab]	**0.561**	**0.195**	**0.267**	**0.198**
AIP	SA-MTP[a]	0.796	0.577	0.672	0.636
	SA-MTP[ab]	**0.797**	**0.584**	**0.680**	**0.644**
AMP	SA-MTP[a]	0.734	0.468	0.757	0.727
	SA-MTP[ab]	**0.745**	**0.492**	**0.765**	**0.742**
APP	SA-MTP[a]	0.520	0.069	0.042	0.061
	SA-MTP[ab]	**0.527**	**0.109**	**0.049**	**0.090**
AVP	SA-MTP[a]	0.736	0.497	0.588	0.555
	SA-MTP[ab]	0.729	**0.500**	**0.591**	**0.556**
CCC	SA-MTP[a]	0.798	0.606	0.651	0.613
	SA-MTP[ab]	**0.806**	0.577	**0.658**	0.584
CPP	SA-MTP[a]	0.741	0.481	0.503	0.505
	SA-MTP[ab]	**0.757**	**0.526**	**0.527**	**0.546**
PBP	SA-MTP[a]	0.686	0.394	0.427	0.394
	SA-MTP[ab]	**0.699**	**0.517**	**0.503**	**0.492**

[ab] The model uses the FiLM classification head.
